# The effectiveness of a co-management care model on older hip fracture patients in China – A multicentre non-randomised controlled study

**DOI:** 10.1016/j.lanwpc.2021.100348

**Published:** 2021-12-31

**Authors:** Jing Zhang, Minghui Yang, Xinyi Zhang, Jiusheng He, Liangyuan Wen, Xianhai Wang, Zongxin Shi, Sanbao Hu, Fengpo Sun, Zishun Gong, Mingyao Sun, Qiang Li, Ke Peng, Pengpeng Ye, Ruofei Ma, Shiwen Zhu, Xinbao Wu, Ruth J Webster, Rebecca Q Ivers, Maoyi Tian

**Affiliations:** a School of Population Health, University of New South Wales, Sydney, New South Wales, Australia; b Department of Orthopaedics and Traumatology, Beijing Jishuitan Hospital, Peking University Fourth School of Clinical Medicine, Beijing, China; c The George Institute for Global Health at Peking University Health Science Centre, Beijing, China; d Department of Orthopaedics, Beijing Shunyi District Hospital, Beijing, China; e Department of Orthopedics, Beijing Hospital, National Center of Gerontology; Institute of Geriatric Medicine, Chinese Academy of Medical Sciences, P.R.China; f Department of Orthopaedics, Beijing Changping District Hospital, Beijing, China; g Department of Orthopaedics, Beijing Liangxiang Hospital, Beijing, China; h Department of Orthopaedics, Beijing Anzhen Hospital, Capital Medical University, Beijing, China; i The George Institute for Global Health, University of New South Wales, Sydney, New South Wales, Australia; j National Clinical Research Center for Cardiovascular Diseases, Shenzhen, Fuwai Hospital Chinese Academy of Medical Sciences, Shenzhen, Shenzhen, China; k National Centre for Non-Communicable Disease Control and Prevention, Chinese Centre for Disease Control and Prevention, Beijing, China; l Centre for Health Economics Research and Evaluation, University of Technology Sydney, Sydney, Australia; m School of Public Health, Harbin Medical University, Harbin, China

**Keywords:** Hip fracture, Co-management care, Orthogeriatric care, Older people, China

## Abstract

**Background:**

Clinical guidelines recommend orthogeriatric care to improve older hip fracture patients’ outcomes, but few studies have been conducted in China. This study evaluated the effects of an orthogeriatric co-management care model in six Chinese hospitals.

**Methods:**

This non-randomised controlled study was designed as an exploratory trial and was conducted in 3 urban and 3 suburban hospitals. Eligible patients were aged ≥ 65 years with X-ray confirmed hip fracture and admitted to hospital within 21 days of injury. All patients received three times follow-ups within one year (1-month, 4-month and 12-month post admission). Co-management care was implemented in 1 urban hospital, while usual care continued in 5 urban and suburban hospitals. Patient demographics, pre-, peri- and post-operative information, complications and mortality were collected at baseline and follow-ups. The primary outcome was proportion of patients receiving surgery within 48 hours from ward arrival. Secondary outcomes included osteoporosis assessment, in-hospital rehabilitation, length of hospital stay, in-hospital mortality and one-year cumulative mortality.

**Findings:**

There were 2,071 eligible patients enrolled (1,110 intervention, 961 control). Compared to usual care, a significantly higher proportion of intervention patients received surgery within 48 hours (75% vs 27%, p<0.0001), osteoporosis assessment (99.9% vs 60.6%, p<0.0001), rehabilitation (99.1% vs 3.9%, p<0.0001) and shorter length of hospital stay (6.1 days vs 12.0 days, p<0.0001). The intervention group saw a significant lower in-hospital mortality rate than the control group (adjusted relative risk 0.021, 95% CI 0.001 to 0.45, P=0.01). One-year cumulative mortality was also significantly reduced in the intervention group (hazard ratio 0.59, 95% CI 0.38 to 0.80, p=0.01).

**Interpretation:**

Co-management care of older hip fracture patients resulted in better outcomes, including decreased time to surgery, improved clinical management, and reduced one-year mortality. A randomised controlled trial is needed to provide definitive evidence.

**Funding:**

The study is supported by Capital's Funds for Health Improvement and Research (2018-1-2071).

**Research in Context**

**Evidence before this study**

Many guidelines in high-income countries recommend the orthogeriatric co-management care for better management of older hip fracture patients. A previous pilot pre- and post- orthogeriatric co-management care in China, involving multidisciplinary team composed of ED physician, anaesthesiologists, radiologists, and physiotherapists, saw significant effects on the proportion of patients who receiving surgery within 48 hours from admission to the orthogeriatric ward (OR=14.9, 95% CI 11.7 to 18.7; p<0.0001), as well as on an improvement of other clinical outcomes. However, this single-centre, retrospective study without parallel controls and follow-up data limited the scope of interpreting the research findings.

**Added value of this study**

This exploratory, multicentre, quasi-experimental study with a paralleled control group was to test the effect of the orthogeriatric co-management care between the intervention and control groups. Compared to the control group, we identified a statistically significant increase on the proportion of patients who receiving the surgery within 48 hours from admission to the ward (75% vs 27%, p<0.0001), osteoporosis assessment (99.9% vs 60.6%, p<0.0001) and rehabilitation (99.1% vs 3.9%, p<0.0001). Additionally, in-hospital mortality and one-year cumulative mortality were significantly reduced in the intervention group.

**Implication of all the available evidence**

This is the first multicentre, prospective, controlled study to evaluate the effect of an orthogeriatric co-management care model in China. This care model significantly reduced the time from ward admission to surgery, improved many outcomes for better hip fracture management as well as showed a promise to reduce in-hospital and one-year cumulative mortality. Despite non-randomisation, the findings provided an important evidence for the scale-up of the orthogeriatric co-management care model in the future.

Alt-text: Unlabelled box

## Introduction

Hip fracture is a common and severe injury among older people, particularly those with existing osteoporosis, imposing a huge burden on patients and health systems, due to high mortality, severe disability, loss of independence, long hospital stay, and excess medical costs.^1^, ^2^, ^3^ The UK “Blue Book”, jointly developed by the British Orthopaedic Association and British Geriatric Society, is considered as the best practice for the management of older hip fracture patients in many countries.^4^ The “Blue Book” outlines six standards for high-quality hip fracture care, including admission to the orthopaedic ward within 4 hours of patients’ presentation, receiving surgery within 48 hours from admission to the ward, minimisation of the risk of a pressure ulcer development, receiving early orthogeriatric involvement, anti-osteoporosis treatment and falls prevention.^4^ A meta-analysis demonstrated that surgery within 48 hours of admission significantly reduced mortality risk in hip fracture patients (OR=0.74, 95% confidence interval [CI] 0.67 to 0.81),^5^ while another study showed that orthogeriatric care was associated with a significant reduction of in-hospital mortality (RR=0.60, 95% CI 0.43 to 0.84).^6^ In addition, early in-hospital rehabilitation improved the quality of life for hip fracture patients.^7^

Despite well-established clinical guidelines for older hip fracture patients, the implementation of those best practices in China remains limited.^8^ In 2015, a hip fracture co-management program, involving orthopaedics and geriatricians, was piloted and evaluated in Beijing Jishuitan Hospital.^9^ The study identified that co-management care significantly increased the proportion of patients who received surgery within 48 hours of ward admission (OR=14.9, 95% CI 11.7 to 18.7; p<0.0001), reduced the development of pressure ulcers (OR=0.3, 95% CI 0.1 to 0.7; p=0.009), and improved geriatrician engagement.^9^ However, this single-centre, retrospective, “pre- and post-” study without parallel controls and follow-up data limited the scope of interpreting the research findings.

We therefore designed and implemented this multicentre prospective non-randomised controlled study, with the aim to evaluate a co-management care model of older hip fracture patients in China on the quality standards guided by the UK “Blue Book”,^4^ compared with usual care.

## Methods

### Study design and settings

This study was designed as an exploratory multicentre quasi-experimental study with non-randomised controls, and was conducted in six hospitals in Beijing, China, including three urban hospitals (Beijing Jishuitan Hospital [JST], Beijing Hospital [BJ], Anzhen Hospital [AZ]), and three district-level suburban hospitals (Beijing Changping District Hospital [CP], Beijing Shunyi District Hospital [SY], and Beijing Liangxiang Hospital [LX]). All hospitals admitted patients from both urban and rural areas. Ethics approvals were received from the Institutional Review Board at Peking University Health Science Centre (IRB00001052-17021) and Biomedical Ethics Committee at Beijing Jishuitan Hospital (201807-11). All informed participants provided written consent. The study was registered at Clinicaltrials.gov (NCT03184896). The study was carried out in accordance with the Helsinki Declaration.

### Study population, recruitment and follow-up

Included patients were aged 65 and older with X-ray confirmed hip fracture (intracapsular, intertrochanteric and subtrochanteric fracture) and were admitted into the study hospitals within 21 days of the fractures. Patients with pathological (tumour) or periprosthetic fractures or terminal malignancies were excluded.

All hip fracture patients who presented to the six hospitals were screened and those who met the inclusion criteria were included in the study. Recruitment of patients commenced from November 26^th^, 2018, while the last patient follow-up was completed on November 30^th^, 2020. All eligible patients were continuously enrolled in the first year (from November 26^th^, 2018 to November 25^th^, 2019). Enrolled patients were then followed up at three time points via telephone (30 days, 120 days, and one-year post admission).

### Intervention and control

The essential elements of the intervention were the establishment of the orthogeriatric ward, and the implementation of the co-management care model started from the time of admission to the Emergency Department (ED) to discharge from the hospital.^9^ In the ED, patient care was jointly provided by the orthopaedic surgeons and ED physicians, with the participation of anaesthesiologists. The ED physicians provided assessments including electrocardiogram and blood tests. Immediately after the patients were admitted to the orthogeriatric ward, orthopaedic surgeons and geriatricians jointly led the care of the patients. Geriatricians saw patients everyday including weekdays and weekends, and provided pre-operative assessment, comorbidity treatment, post-operative prevention of complications and secondary prevention of fracture (i.e., bone protection and falls assessment). In the orthogeriatric ward, nutritionists, physiotherapists and nurses were also involved in the pre-, peri- and post-operative assessment and treatment. The details of the patient journey were described elsewhere.^10^ The co-management care adapted the recommendations of the UK guideline in hip fracture management, including quick admission to an orthogeriatric ward, expedited surgery, geriatrician assessment, secondary prevention of fracture, pressure ulcer prevention, provision of physiotherapy, and early discharge.^11^ The intervention was implemented in JST hospital throughout the study period. All patients enrolled in JST hospital received the co-management care.

The other five participating hospitals continued their usual care (control arm). Patients in the control arm were admitted to the orthopaedic ward. The usual care was mainly provided by the orthopaedic surgeon, while there was unscheduled internal physician or geriatrician consultation if required.

### Data collection

Trained nurses from the orthopaedic ward in each hospital were responsible for patients’ screening, enrolment, and data collection at the baseline and follow-ups. The baseline data included patient demographic information (e.g., age and gender), pre-operative information (e.g., quality of life,^12^ comorbidity and pre-fracture mobility), peri-operative information (e.g., if performing surgery, type of surgery and anaesthesia) and post-operative information (e.g., full weight bearing and psychological support to reduce fear of falling), while the follow up information contained information of mobility, complications, and mortality. Data were collected using a tablet-based Research Electronic Data Capture (REDCap) system.

### Study outcomes

Primary and secondary outcomes were selected primarily based on the important process outcomes of the quality standards outlined in the UK “Blue Book” and the clinical outcomes of interests.^4^ The primary outcome was the proportion of patients who received surgery within 48 hours from ward arrival. Secondary outcomes within the “Blue Book” quality standards included: the proportion of patients who were admitted to orthopaedic wards within 4 hours from the ED arrival; the proportion of patients receiving osteoporosis assessment in hospital; the proportion of patients developing pressure ulcers in hospital; and the proportion of patients receiving falls assessment in hospital. Other secondary outcomes were: the proportion of patients receiving surgery within 48 hours from the ED arrival; the proportion of patients receiving rehabilitative care before discharge; the length of hospital stay; in-hospital mortality; and one-year cumulative mortality after hip fracture, including in-hospital mortality.

### Statistical Analysis

All participants’ demographic and clinical characteristics at baseline and follow-up were tested between the intervention and control group, using Chi-square test for categorical variables and t-test for continuous variables with two-sided p values. The primary outcome and binary secondary outcomes were compared between two groups using log-binomial regression models, while the continuous secondary outcomes were compared using multivariable linear regression models. Both models were adjusted for participants’ demographic and clinical characteristics as potential confounders. The potential confounders were defined as either those variables with p-value ≤ 0.1 from univariable analysis (Table 1) or clinical relevance, including demographic information, pre- and/or peri- and/or post-operative characteristics.

**Table 1:** Patient demographic and clinical characteristics

**Characteristics**	Intervention	Control						P value*
	JST(N=1,110)	BJ (N=223)	AZ (N=82)	CP (N=191)	SY (N=282)	LX (N=183)	Subtotal (N=961)	
**Demographics**								
Age (years), Mean (SD)	79.7 (7.8)	80.6 (6.6)	81.9 (7.6)	79.0 (7.3)	79.5 (7.5)	79.1 (7.3)	80.0 (7.4)	0.5
Female (%)	72.1	66.4	76.8	55.5	60.6	71.0	56.3	0.0001
Illiterate (%)	18.2	17.5	15.9	29.8	32.6	29.0	26.4	<0.0001
Having health insurance available (%)	84.1	95.1	95.1	87.4	98.6	91.8	94.0	<0.0001
Living in rural areas (%)	8.3	3.1	6.1	66.0	74.1	53.0	46.2	<0.0001
Living alone (%)	11.8	13.9	4.9	10.4	10.3	8.7	10.7	0.45
**Pre-operative characteristics**								
Utility index scores of EQ5D (SD)	0.108 (0.188)	0.289 (0.215)	0.361 (0.347)	0.357 (0.302)	0.128 (0.181)	0.203 (0.182)	0.243 (0.252)	<0.0001
Housebound pre-fracture (%)	23.5	21.1	31.7	25.1	16.7	38.3	24.8	0.49
Without aids post-fracture (%)	2.3	2.2	1.2	20.4	6.0	1.6	6.8	<0.0001
Having cognitive impairment (%)	10.4	26.0	25.6	6.8	71.3	16.9	33.7	<0.0001
With comorbidities (%)	12.5	14.4	32.9	27.2	5.0	17.5	16.3	0.01
Femoral neck fracture (%)	51.3	53.8	48.8	32.4	10.6	43.2	34.4	<0.0001
Regional anaesthetic blocks for pain management in the ED (%)	70.1	33.2	52.4	7.9	97.9	36.6	49.4	<0.0001
**Peri-operative characteristics**								
Received operation (%)	98.3	90.6	81.7	86.4	98.9	80.9	89.6	<0.0001
General anaesthesia (%)	2.2	4.0	4.5	1.2	28.3	2.7	11.2	<0.0001
Intramedullary nailing fixation (%)	48.6	50.5	47.8	66.7	90.3	59.5	67.8	<0.0001
**Post-operative characteristics**								
Full weight bearing on the day post-surgery (%)	43.5	21.3	32.8	4.9	17.2	33.9	19.9	<0.0001
Psychological support to reduce fear of falling (%)	86.3	13.9	22.0	39.3	34.4	3.3	23.6	<0.0001
Mobility without aids pre-discharge (%)	2.6	0	3.7	2.6	0	0	0.8	<0.0001
**Post-discharge characteristics**								
Having reoperation post-discharge (%)	1.3	0.9	0	2.1	0.7	2.2	1.3	0.99
Having complications post-discharge (%)	8.8	9.2	21.3	7.0	9.0	35.9	14.8	<0.0001
Having anti-osteoporotic medicine post-discharge (%)	86.0	77.5	100	17.7	36.2	82.3	56.3	<0.0001
Receiving rehabilitation post-discharge (%)	69.2	46.2	41.5	36.1	95.4	86.3	65.9	0.1

*P-value between intervention and control groups.

The one-year cumulative mortality was defined as a death occurring within one-year from the ward admission (370 days as the upper range of follow-up) in two groups. The Kaplan-Meier curve estimated the unadjusted survival probability against time. The cox proportional hazards regression model was performed for the hazard ratio (HR) of one-year cumulative mortality rate between the intervention and control groups adjusting all potential confounders used for the primary outcome plus post-discharge characteristics.

### Role of the funding source

The funder had not influenced the study design, data collection, data analysis, interpretation, and writing of this manuscript.

## Results

### Study flow chart

**Figure 1:**
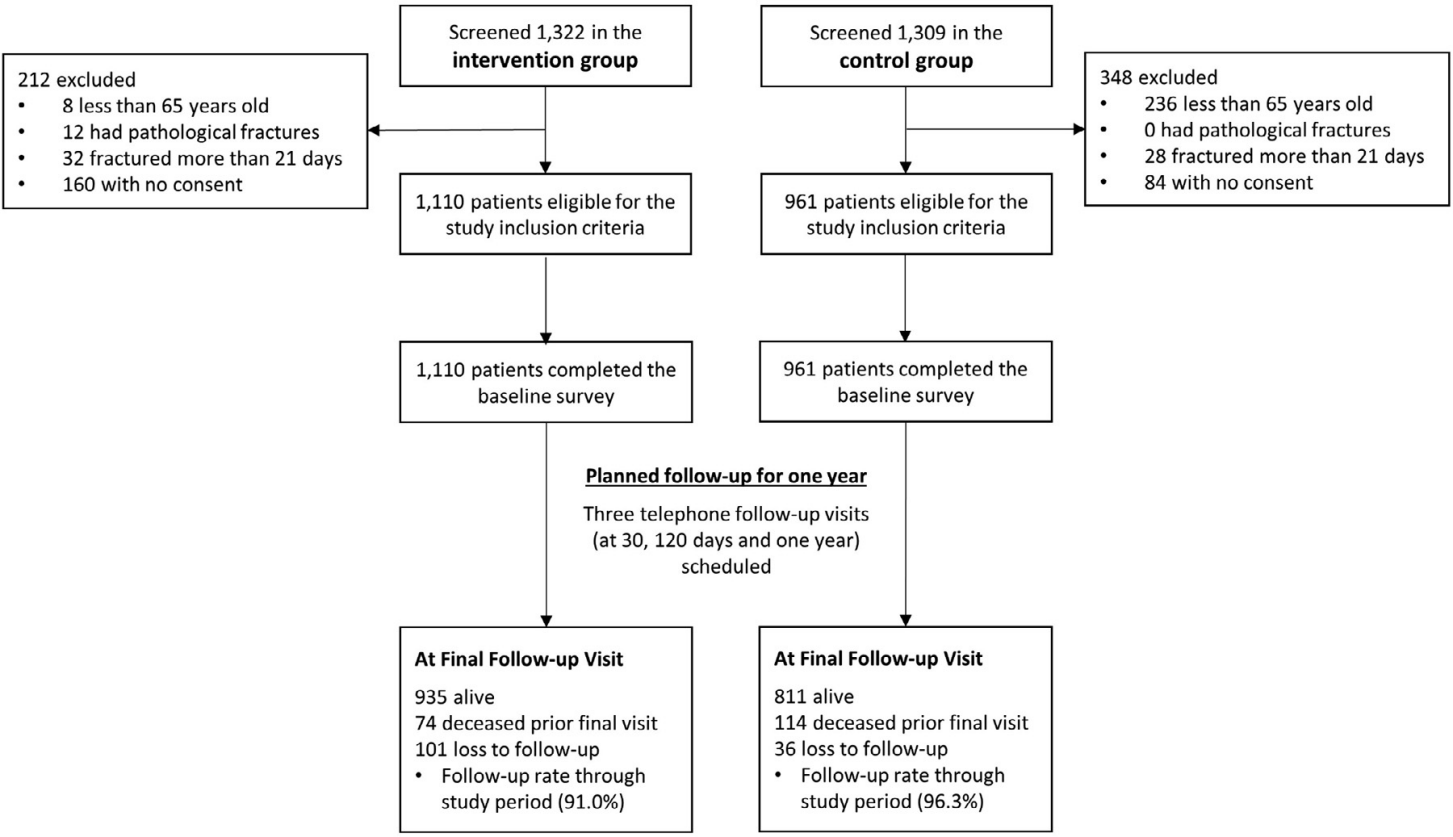
Flowchart of Patient Enrolment.

A total of 2,631 patients were admitted across the six hospitals over one year. Of those, 560 patients were excluded with reasons given in Figure 1. There were 2,071 patients enrolled in this study, with 1,110 and 961 patients in the intervention and control groups respectively.

### Patient characteristics

The selected characteristics of enrolled patients are shown in Table 1. The average age of all patients was 80 years old. In comparison with the intervention group, the patients in the control group had a significantly higher proportion of males (43.7% versus 27.9%; p=0.0001), patients who were illiterate (26.4% versus 18.2%; p<0.0001), rural residents (46.2% versus 8.3%; p<0.0001), cognitive impairment (33.7% versus 10.4%; p<0.0001), and comorbidities (16.3% versus 12.5%; p=0.01) before surgery. After discharge, the control group had a higher rate (14.8% versus 8.8%; p<0.0001) of complications (delirium, pneumonia, deep venous thrombosis, etc.) and lower rate of receiving anti-osteoporotic medicines (56.3% versus 86.0%; p<0.0001). Patients in the intervention group had a significantly higher proportion of femoral neck fracture (51.3% versus 34.4%; p<0.0001), regional anaesthetic blocks for pain management in the ED (70.1% versus 49.4%; p<0.0001), receiving an operation (98.3% versus 89.6%; p<0.0001), and early full weight bearing (43.5% versus 19.9%; p<0.0001).

**Figure 2:**
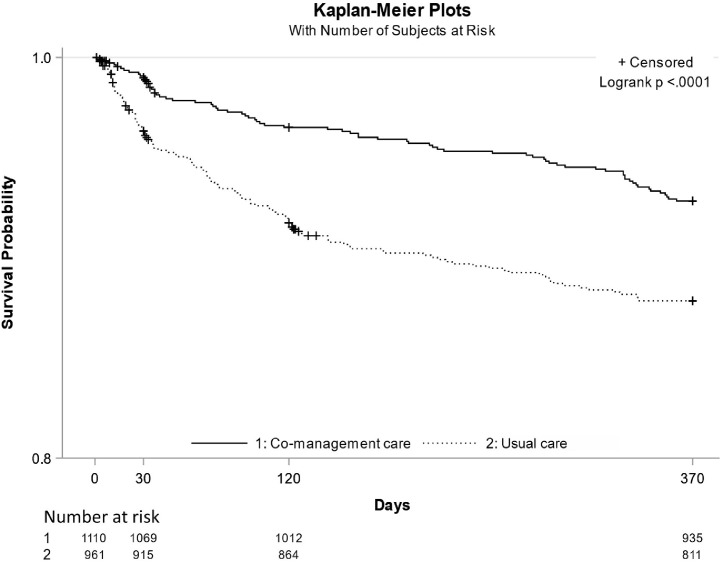
One-year Survival Probability in Intervention and Control Groups.

For the primary outcome, in the intervention group, approximately three quarters of patients received an operation within 48 hours after admission to the orthogeriatric ward, a result statistically significantly higher than the control group (75.3% versus 27.3%, RR=2.7, 95% CI 2.4 to 3.0; p<0.0001) after adjusting for all confounders. For the secondary outcomes, similarly, compared to the control group, a higher proportion of patients in the intervention group had an operation within 48 hours from ED arrival (42.9% versus 23.3%, RR=2.0, 95% CI 1.8 to 2.3; p<0.0001) after adjusting for all confounders. The intervention group were less likely to be admitted within 4 hours to the orthogeriatric ward from the ED (5.1% versus 87.1%, RR=0.48, 95% CI 0.44 to 0.51; p<0.0001), but had shorter length of hospital stay (6.1 versus 12.0 days, mean difference = -6.7, 95% CI -7.6 to -5.8; p<0.0001) and lower in-hospital mortality (0.1% versus 1.7%, RR=0.021, 95% CI 0.001 to 0.45; p=0.01) after adjusting for all confounders. Almost all patients received osteoporosis treatment and early rehabilitation before their discharge in the intervention group, whereas these outcomes were statistically significantly lower in the control group (all p<0.0001). The effects of co-management care on the primary and secondary outcomes are shown in Table 2. There was a statistically significantly lower incidence rate of one-year cumulative mortality in the intervention group compared to the control group which would also be considered clinically significant (7.3% versus 12.3%, HR=0.59, 95% CI 0.38 to 0.80, p=0.01) as shown in Figure 2.

**Table 2:** Effects on the primary and secondary outcomes

Outcomes	Intervention (N=1,110)	Control (N=961)	Relative Risk / mean difference (95%CI)	P value	Adjusted Relative Risk / mean difference (95%CI)	Adjusted P value
**Primary outcome**						
Surgery within 48 hours from admission to ward (%)*	75.3	27.3	2.8 (2.5 – 3.1)	<0.0001	2.7 (2.4 – 3.0)	<0.0001
**Secondary outcomes**						
Surgery within 48 hours from ED arrival (%)*	42.9	23.3	1.8 (1.6 – 2.1)	<0.0001	2.0 (1.8 – 2.3)	<0.0001
Less than 4 hours from emergency to ward arrival (%)*	5.1	87.1	0.058 (0.045 – 0.075)	<0.0001	0.48 (0.44 – 0.51)	<0.0001
Receiving osteoporosis assessment in hospital (%)#	99.9	60.6	NA	<0.0001	NA	<0.0001
Receiving rehabilitation before discharge (%)#	99.1	3.9	NA	<0.0001	NA	<0.0001
Developing pressure ulcers in hospital (%)&	3.0	1.8	1.7 (0.9 – 3.0)	0.08	1.04 (0.362 – 2.927)	0.95
Receiving falls assessment in hospital (%)&	98.8	97.6	1.01 (1.00 – 1.02)	0.04	0.967 (0.837 – 1.009)	0.49
Length of hospital stay, days (SD)&	6.1 (5.3)	12.0 (9.5)	-5.9 (-6.53 – -5.23)	<0.0001	-6.7 (-7.6 – -5.8)	<0.0001
In-hospital mortality (%)&	0.1	1.7	0.05 (0.01, 0.41)	<0.0001	0.021 (0.001, 0.45)	

*Adjusted confounders include sex, education level, having insurance or not, living setting, living status, utility index scores of EQ5D, pre-operative mobility, having cognitive impairment or not, having comorbidities or not, type of fracture, and having regional anaesthetic blocks for pain management in ED;

# Adjusted confounders include * + received surgery, type of anaesthesia, and type of surgery;

& Adjusted confounders include * + # + full weight bearing on the day post-surgery, psychological support to reduce fear of falling, and pre-discharge mobility.Primary and secondary outcomes

## Discussion

This study evaluated the effectiveness of a co-management care model for older hip fracture patients in six Chinese hospitals. Compared to usual care, co-management care involving orthopaedics and geriatricians significantly improved two quality standards of the best practice for hip fracture management outlined in the UK “Blue Book”. In addition, the co-management intervention reduced the risk of in-hospital and one-year cumulative mortality, although this requires more robust confirmation in a larger randomised trial.

In this study, about three quarters of patients received surgery within 48 hours post ward admission under co-management care, while in the control group, the proportion of patients receiving surgery within the recommended timeframes remains low. The significant difference in time to surgery may be due to early optimal management of comorbidities before surgery, and better coordination of care in the intervention group. There was an additional improvement seen when compared to the previous pilot study conducted in JST hospital in 2017, where the proportion of patients having surgery within 48 hours was around 60%.^9^ Further, the 75% of patients who received surgery within 48 hours was comparable to that found in the UK in 2019 at 68%.^13^ Expedited surgery for older hip fracture patients is promoted in many countries’ guidelines,^4^,^11^,^14^,^15^ to alleviate pain, facilitate early mobilisation, and reduce the risks of distress, morbidity, and mortality.^16^,^17^ The reasons for delayed surgery are complex and may include unnecessary pre-operative examinations, significant elongated consultation time, poor communication and coordination between clinicians and departments.^18^,^19^ Under co-management care, unlike the usual care, the orthopaedics and geriatricians have shared responsibilities, exchanged patients’ information in a timely manner to accelerate the logistics required for the surgery, and proactively engaged with other departments that play a role in the management of hip fracture.^20^,^21^ This aligns with the integrated care model for older patients approach, advocated by the World Health Organisation, to improve the engagement and coordination of different health care providers within or across the health facilities.^22^

It was evident from this study that involvement with geriatricians for older hip fracture patients substantially improved the post-surgery falls assessment, osteoporosis assessment, and physiotherapy. However, in the control groups, osteoporosis assessment and physiotherapy were less provided for patients. This was primarily because either a geriatrician was not available or was not involved in the management of hip fracture. In contrast with western countries, geriatric medicine is under-developed in China, with only approximate one quarter of hospitals above primary health care level being equipped with a geriatric medicine department in Beijing in 2018.^23^,^24^ However, as an alternative in JST hospital, ED physicians fulfilled the role of geriatrician to provide all required assessment in the ED. The appropriate involvement of geriatricians appears to be the critical element of the co-management care model. The guideline in the UK particularly emphasised on involving geriatrician assessment and management in the perioperative period, postoperative geriatrician-led rehabilitation, and secondary prevention of fracture during the management of older hip fracture patients.^11^ Evidence showed that collaboration between orthopaedics and geriatricians can also significantly reduce patient mortality at discharge and follow-up, hospital length of stay and risk of delirium, and improve functional outcomes through joint efforts during post-surgery care.^6^,^25^

In this study, we identified that only a small proportion (5.1%) of patients in the JST hospital was admitted to the ward from ED within 4 hours, which was significantly lower than the control group. Despite the delays in the ED, the intervention group still saw a significantly higher proportion of patients receiving surgery within 48 hours from ED arrival (42.9%) than that in the control group (23.3%). This was predominantly due to an insufficient number of beds in the orthogeriatric ward, limiting the availability of ward arrival within the recommended timeframe.^9^ In the intervention group, in fact patients were managed by the ED physicians for their comorbidities prior to the ward admission. However, there was no evidence on the association between ward arrival time and patient's health outcomes. This suggested that the implementation of co-management care focused on the integration of different health providers, regardless of where the intervention was provided, i.e. ED or ward.

Compared to the control group, the in-hospital mortality and one-year mortality in the intervention group were 0.1% and 7.3%, significantly reduced by 94% and 41%, respectively. We hypothesise that the co-management care might have had a positive impact on patients’ survival in the acute and post-acute phases, possibly due to expedited surgery and better management of the complications post-surgery. Previous studies demonstrated similar findings that integrated orthogeriatric care was an effective way to promote best practice in hip fracture management, and can significantly reduce the in-hospital and one-year mortality.^26^, ^27^, ^28^, ^29^ However, these findings require confirmation in an appropriately designed randomised trial. Additionally, no randomised controlled trials are currently designed to robustly evaluate effectiveness of the orthogeriatric co-management care model in resource-constrained settings, and there is an urgent need to develop a confirmatory trial to provide definitive evidence.

One of the study strengths was the prospective study design reducing recall bias during the data collection and ensuring internal validity. In addition, multicentre settings and high follow-up rates ensured sufficient sample size and accurate outcome measurements for evaluation. There are several limitations to this study. First, despite efforts were made to adjust for all confounders in the analysis, the non-randomisation of the intervention and control groups may induce selection bias into the study. Second, the intervention hospital was better resourced in infrastructure, compared to the control groups. The difference of infrastructure was not adjusted in the analysis. Third, patients’ severity of comorbidities, a strong predicator of certain study outcomes, was not collected. Fourth, although this study was designed as a prospective study, the participants were asked to provide information about study outcomes via recall at each scheduled telephone follow-up. This may have resulted in recall bias. Lastly, this study was only conducted in Beijing, a well-developed city in China. The effectiveness of the intervention in resource-constrained settings is therefore unknown.

## Conclusion

To our knowledge, this is the first multicentre, prospective, controlled study to evaluate the effectiveness of an orthogeriatric co-management care model in China. This care model significantly reduced the time from ward admission to surgery and improved many process outcomes for better hip fracture management, such as access to osteoporosis treatment and rehabilitation. Despite the study results showing promise to improve patients’ mortality outcome, a randomised controlled trial is needed for a definitive outcome.

### Contributors

JZ is first author who contributed to the first draft. MY, MT and JZ designed the study. MT, JZ and KP developed the questionnaire. JZ and XZ were responsible for data collection and data cleaning. JZ and QL was responsible for the data analysis and interpretation. MY, RI, RW and MT contributed to critical revision of the manuscript. All authors participated in the study management.

### Data sharing statement

The dataset is managed by Beijing Jishuitan Hospital. The data access request can contact the corresponding author.

## Declaration of interests

All authors have not any conflict of interest to this study and have provided a conflict of interest statement.
